# Using an ontology of the human cardiovascular system to improve the classification of histological images

**DOI:** 10.1038/s41598-020-69037-4

**Published:** 2020-07-23

**Authors:** Claudia Mazo, Enrique Alegre, Maria Trujillo

**Affiliations:** 1 UCD School of Computer Science, University College Dublin, Dublin, Ireland; 2 CeADAR: Centre for Applied Data Analytics Research, Dublin, Ireland; 3 Industrial and Informatics Engineering School, Universidad de León, León, Spain; 4 Computer and Systems Engineering School, Universidad del Valle, Cali, Colombia

**Keywords:** Preclinical research, Biomedical engineering

## Abstract

The advantages of automatically recognition of fundamental tissues using computer vision techniques are well known, but one of its main limitations is that sometimes it is not possible to classify correctly an image because the visual information is insufficient or the descriptors extracted are not discriminative enough. An Ontology could solve in part this problem, because it gathers and makes possible to use the specific knowledge that allows detecting clear mistakes in the classification, occasionally simply by pointing out impossible configurations in that domain. One of the main contributions of this work is that we used a Histological Ontology to correct, and therefore improve the classification of histological images. First, we described small regions of images, denoted as blocks, using Local Binary Pattern (LBP) based descriptors and we determined which tissue of the cardiovascular system was present using a cascade Support Vector Machine (SVM). Later, we built Resource Description Framework (RDF) triples for the occurrences of each discriminant class. Based on that, we used a Histological Ontology to correct, among others, “not possible” situations, improving in this way the global accuracy in the blocks first and in tissues classification later. For the experimental validation, we used a set of 6000 blocks of $100\times100$ pixels, obtaining F-Scores between 0.769 and 0.886. Thus, there is an improvement between 0.003 and $0.769\%$ when compared to the approach without the histological ontology. The methodology improves the automatic classification of histological images using a histological ontology. Another advantage of our proposal is that using the Ontology, we were capable of recognising the epithelial tissue, previously not detected by any of the computer vision methods used, including a CNN proposal called HistoResNet evaluated in the experiments. Finally, we also have created and made publicly available a dataset consisting of 6000 blocks of labelled histological tissues.

## Introduction

A well-known research problem, in the histological context, consists of the recognition of patterns and infer the organisation of the fundamental tissues which enable the identification of specific organs. To recognise fundamental tissues on histological images, healthy tissues, automatically may be helpful to reinforce the learning process of histologists, biologists, pathologists, and those in related disciplines^1^. A medicine student must first learn to recognise healthy fundamental tissues prior to identifying pathological tissue. Laboratory practices require an expert to corroborate that students learn as expected to identify tissues and organs in different histological samples. However, laboratory practices are time and frequency limited. Also, an expert’s personalised verification per student depends on the number of students and laboratory practice duration. An automated tissue recognition may increase the number of cases that a student may analyse, promoting self-learning to on-campus students and also facilitating on-line learning to external or remote students through E-Learning systems^2^. Additionally, automatic identification of histological images solves some problems present in manual annotation, such as subjectivity, time costs, difficult, and impracticality^3^. On the other hand, a histological ontology is a knowledge representation which makes possible to describe information about the real world in a form such that a computer system can use it to solve complex tasks as (i) communicate specifically, clearly and precisely histology concepts and (ii) represent or model knowledge from histology data sources in order to interact and process it automatically. However, the different types or representations of data that are obtained cannot be treated in the same way, what is an open problem. In this work, we present a new method to classify histological tissues using both image descriptors and the knowledge obtained from an ontology of the human cardiovascular system. We use the histological ontology to improve the classification based on image descriptors achieving in this way lower margins of error or uncertainties than those obtained when using a single information source. This work constitutes a new way to perform multiple heterogeneous information integration in the histological context in order to identify patterns and infer the organisation of the fundamental tissues.

In histological context^4^ proposes to leverage the high-level reasoning and knowledge formalisation ability of ontology-based software to make annotation of high-content images more efficient and interactive. The low-level image processing aims at outlining and describing general biological objects in the histopathological images. In this step, thresholding, morphological closure and snake-based method are used. They use an anatomical ontology to refine the specificity and sensibility rate using SPARQL query language. The results show that the proposed method detected all mitoses but the detection has many false positive. However, this work is focused in histopathological images and anatomical ontology. Smith et al.^5^ outline the main difficulties, particularly faced by ontologies in the fields of histopathological imaging and image analysis, and suggests a strategy for addressing these challenges. This review presented the use of ontologies in annotation or tagging, investigations and biobanking. Furthermore, a critical survey of major existing contributions to ontologies and ontology-related standards in the imaging domain is provided. However, this review is focused in histopathological imaging. Furthermore, other application domains have used ontologies to improve the classification process using different strategies. Abdollahpour et al.^6^ introduce a new visual word generation and feature representation method for multi-class image classification based on semantic taxonomies. They used visual features of all the sub-concepts modelled in the taxonomy to represent the images as a histogram of visual words’ occurrences. Finally, they used the semantic relation between classes based on WordNet hierarchy and then assign a set of linear SVM to each semantic node. The CIFAR-10 dataset^7^ was used for testing, given better accuracy than the baseline methods of one vs. one, one vs. all and another reference method. The CIFAR-10 is labelled subsets of tiny images dataset which contains 10 classes (airplane, automobile, bird, cat, deer, dog, frog, horse, ship and truck). However, they used a taxonomy which is limited in inferencing potential due to lack of relational expressiveness about its content. Breen et al.^8^ proposed a scalable system capable of examining images and accurately classifying the image based on its visual content combining ontologies and neural networks. Neural networks are used as object identifiers to do the automatic classification of an image based in its content—colour distribution. Ontologies are used to represent relations that reveal useful information in classifying the entire image. After a network identifies a set of objects from an input image, these objects may be used to select concepts from ontologies. The combined system was tested with 15 sample images from the sport domain obtaining that between 33% and $87\%$ of the images are associated with relevant concepts and $13\%$ and $77\%$ images are associated with at least one irrelevant concept according the threshold.

As a part of our proposal, preliminary advances have been done in the identification and classification of fundamental tissues^9–12^ and organs^13–15^ of the human cardiovascular system. Additionally, a histological ontology was created^16^. This work is a part of a Doctoral dissertation from Universidad del Valle (Cali-Colombia), and Universidad de León (León-Spain) in a double degree agreement^17^ and some future works were developed.

In this paper, we describe a methodology to improve the automatic classification of histological images using a histological ontology of the human cardiovascular system. We propose two specific methods: (i) to improve the classification of organs, and (ii) to recognise epithelial tissue using the histological ontology. These methods allow us to increase the F-Score obtained in all cases of the automatic classification of tissues and organs.

The paper is structured as follows: the method to improve the automatic identification is explained in “Methods”; the evaluation and the results are described in “Results”; and some conclusions are presented in “Conclusions”.

## Methods

**Figure 1:**
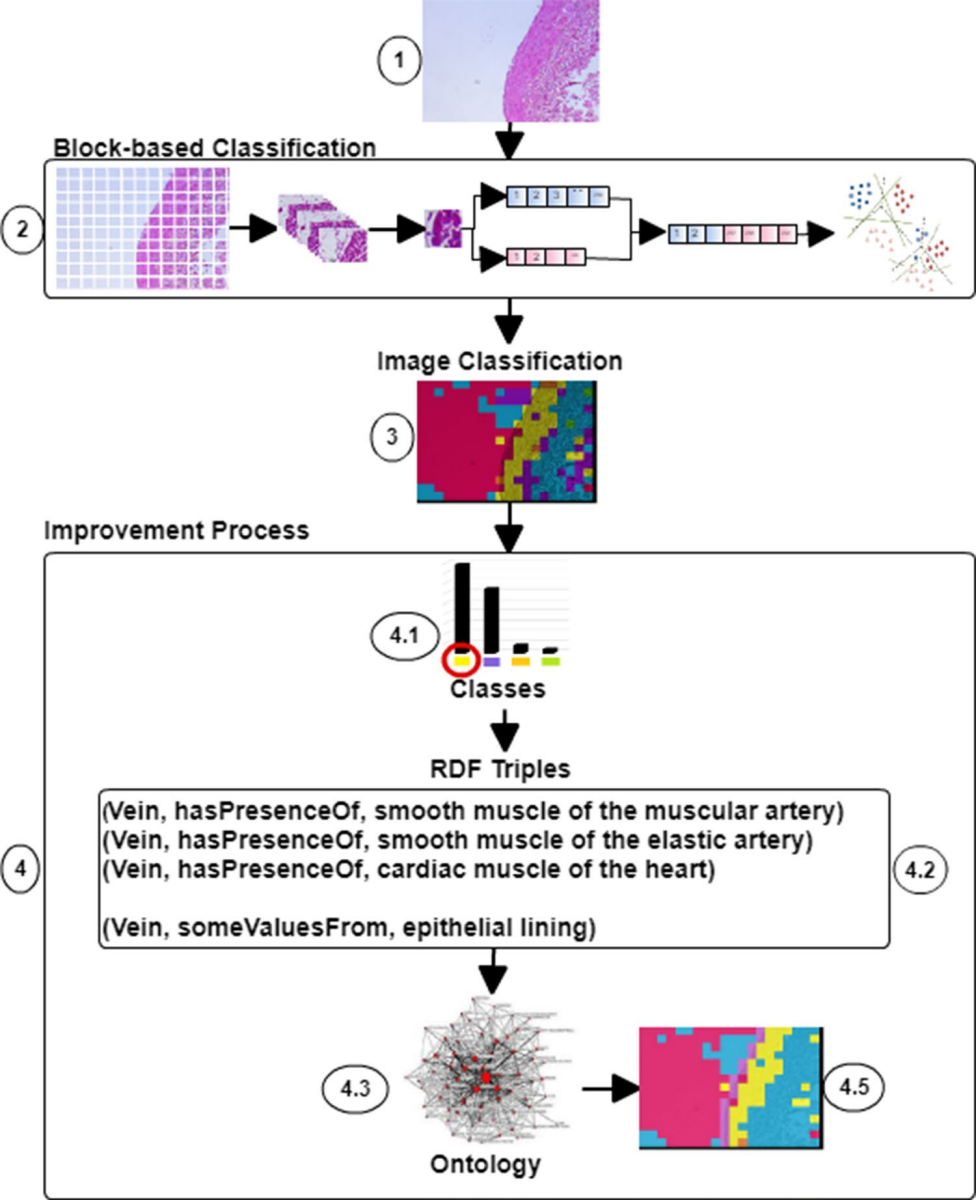
Proposed approach for improving the classification using an example image. (1) Histological image. (2) Classification based on texture features and a cascade SVM^14^. (3) Classification of a histological image. (4) Improvement process. (4.1) Occurrences for each discriminant class. Yellow represent the smooth muscle of the large vein, violet represents the smooth muscle of the muscular artery, orange represents the smooth muscle of the elastic artery, and green represent the cardiac muscle of the heart. (4.2) RDF triples. (4.3) Histological ontology^16^. (4.4) Image’s blocks reclassified.

We propose a methodology to improve the classification of tissues and organs of the human cardiovascular system. A brief summary of the proposed process is given below and a detailed explanation is presented in the following subsections. Figure 1 shows a general outline of our proposal which consists of four steps: (1) a histological image is obtained from a histological sample. (2) Using image processing and machine learning the histological image is classified. In this case, we use LBP based descriptors and a cascade SVM^14^. (3) A histological image is classified based on the tissues recognised in regular patches named blocks. (4) The initial classification is improved based on four steps: (4.1) Occurrences for each discriminant class are calculated. (4.2) RDF^18^ triples are built. (4.3) A histological ontology is used to improve the previous classification results: (i) organ classification is enhanced by reclassifying samples according to the discriminant class with the highest occurrence using RDF; (ii) epithelial tissue classification is improved as it is not identified in 10$\times$ images. (4.4) Image’s blocks are reclassified. The details of the complete process are presented in this section.

### Block-based classification

**Figure 2:**
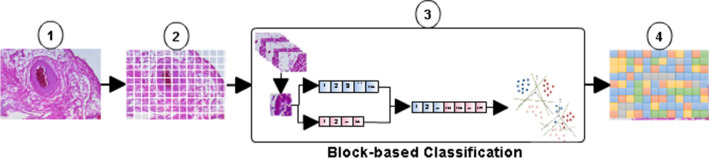
Proposed approach for automatic classification of fundamental tissues and an organ in a histological image: (1) Histological image taken with the $10\times$ objective. (2) Blocks of $100\times100$ pixels. (3) Block-based recognition method. (4) Histological image classification.

We used the approach proposed in^14^ to classify automatically fundamental tissues and, in some cases, the organs of the cardiovascular system by blocks using texture features. Texture feature has been one of the most important and most commonly used low level visual features in content-based medical image^19^. Texture descriptors are used to describe the content of medical images^20^ and histological images^21,22^. Some histological image studies are focused on cell^23,24^ and in pathologies^25–27^. The proposed method in^14^ is three-fold [see Fig. 2(3)]: (i) initially, an image is divided into blocks. Blocks of $100\times100$ pixels are obtained from histological images. In^14^ a block has the following two characteristics: (a) it contains only one type of tissue and (b) it has discriminant information so that tissue recognition is possible. (ii) then, a feature extraction process is performed to obtain relevant information from a block. Information is extracted from blocks using the LBP|LBPri texture descriptor to represent efficiently local micro-patterns. LBP is defined as a gray-scale invariant texture measure. LBP is a method that is used to describe texture characteristics of the surfaces, identifying “uniform” or “non-uniform” texture patterns. LBP is a transformation that maps a current gray level value into a binary representation by comparing the value at a current pixel to each value at its four or eight neighbours^28^:

1 $$\begin{aligned} LBP_{P,R}=\sum _{p=0}^{p-1}{s(g_{p}-g_{c})2^{p}} \qquad s(x)=\begin{Bmatrix} 1,&if&x>=0;\\ 0,&if&x<0; \end{Bmatrix}, \end{aligned}$$

where $g_{p}$ is the gray value of the central pixel and $g_{c}$ is the gray value of the pixel adjacent. LBPri is a modification of LBP, it is achieved by circularly rotating each bit pattern to the minimum value. We use LBP with a radius equal to 1 and 8 neighbours, $LBP_{8}^{ri36}$ (for simplicity, this term is referred to as LBPri henceforth). The feature extraction process returns a feature vector of 292 values obtained by concatenating LBP^28^ (256 values) and LBPri^29^ (36 values). (iii) Finally, LBP|LBPri texture feature vectors are used to first describe and later classify blocks using a SVM^30^. A SVM with a linear kernel is used to classify blocks into one of the following five classes: (1) smooth muscle of the large vein and the elastic artery; (2) smooth muscle of muscular artery; (3) cardiac muscle of the heart; (4) loose connective tissue; and (5) light regions. After that, tissues in the first class are separated again, using another SVM with a polynomial kernel, into: (1.1) smooth muscle of the elastic artery and (1.2) smooth muscle of the large vein.

### Classification of a histological images using block-based recognition

In this subsection we present an approach to classify tissues and organs that appear in a histological image using the block-based recognition method described in the previous subsection. In contrast to the block-based method, this approach includes blocks which may contain one or more type of tissues. A general outline of our proposal is illustrated in Fig. 2. (1) A histological image acquired at $10\times$ objective. (2) Blocks of $100\times100$ pixels are obtained from the histological image. (3) Block-based recognition is used to classify blocks as was described in “Block-based classification”. This approach was selected considering it is possible to separate with very high precision (AUC greater than 0.98) the fundamental tissues of the cardiovascular system along with some organs, such as the heart, arteries and veins^14^. (4) A classification of a complete histological image by blocks.

The classification of a histological image using block-based recognition process is defined as:

Let $I: {\mathbb {I}} \times {\mathbb {I}} \rightarrow {\mathbb {R}}^3$ be a histological image of the cardiovascular system in *RGB* colour space; $I_{s} = \{I_{0}, I_{1}, \ldots , I_{K}\}$ be a set of histological images; *B* be a matrix of blocks in which each $B_{ij}$ represents the *j*th block of the image *i*, note that blocks in a histological image may contain more than one tissue; $M_{rbc}(B_{ij})$ be the block-based recognition method described in Ref.^14^ which classified blocks into one of the six following classes: (i) smooth muscle of elastic artery, (ii) smooth muscle of large vein, (iii) smooth muscle of muscular artery, (iv) cardiac muscle of heart, (v) loose connective tissue—vein, arteries and heart—and (vi) light regions; $R_I$ be a $m \times n$ matrix of labels where $m=width\;Image/block\;Size$ and $n=height\;Image/block\;Size$, in our case $20\times15$. Then, classification of a histological image using block-based recognition is:

2 $$\begin{aligned} R_I = \begin{pmatrix} M_{rbc}(B_{11}) &{} M_{rbc}(B_{12}) &{} \cdots &{} M_{rbc}(B_{1n}) \\ M_{rbc}(B_{21}) &{} M_{rbc}(B_{22}) &{} \cdots &{} M_{rbc}(B_{2n}) \\ \vdots &{} \vdots &{} \cdots &{} \vdots \\ M_{rbc}(B_{m1}) &{} M_{rbc}(B_{m2}) &{} \cdots &{} M_{rbc}(B_{mn}) \end{pmatrix} \end{aligned}$$

### Improving the classification using the ontology

Initially, occurrences of each class, taking into account the results of the classification method, are obtained for the histological image. Apart from this, two sets are defined: discriminating and non-discriminant classes. Discriminant classes are tissues associated to an organ, such as cardiac muscle of the heart, smooth muscle of the muscular artery, smooth muscle of the large vein, and smooth muscle of the elastic artery. Non-discriminant classes are not directly associated to an organ, such as loose connective tissue and light regions.

After, RDF triples are built to use the histological ontology to improve the classification using a reasoner [see Fig. 1 (4.2, 4.3 and 4.4)]. A reasoner is a very important tool in developing and using an ontology written in OWL. Automated reasoners such as Pellet, FaCT++, HerMiT, ELK and among other are included in the Protege user interface^31^. Two open source reasoners were used in this proposal—Pellet and FaCT++, Java and C++ based, respectively. A reasoner take a collection of axioms written in OWL and offer a set of operations on the ontology’s axioms (e.g. inference, subsumption, satisfiability, classification, instance retrieval, conjunctive query answering, entailment, consistency, explanation, among others). The Protege-OWL Reasoning API was used in this proposal. We propose two different ways for improving the classification using a histological ontology as follows:

#### Improving organ classification using the histological ontology

In this step, the organ of the discriminant class with higher occurrences is selected as a subject and the other organs are used as objects to build a RDF triple for each object in the form of subject, predicate, and object. We worked with the higher occurrences taking into account the accuracy of our classification method which describes the image content. For instance, in a case in which the cardiac muscle of the heart is the tissue with higher occurrences, three RDFs are constructed using the heart as subject and, as objects, the muscular artery, the large vein, and the elastic artery, respectively.

Rules in RDF form are built taking into account subject and objects obtained in the last step from discriminant classes and the predicate *hasPresenceOf*. In our example the RDFs are: (i) (the heart, hasPresenceOf, the elastic arteries), (ii) (the heart, hasPresenceOf, the large vein), (iii) (large vein, hasPresenceOf, the elastic artery), and (iv) (large vein, hasPresenceOf, the muscular artery).

These rules are used to make a query with the histological ontology’s reasoner, using SPARQL queries^32^, in order to obtain a result. If the obtained answer is empty, then blocks which were classified with the organ used as object in the RDF triple should be reclassified. The new label will be decided according to the behaviour of false positives in the classification process. Otherwise, the initial classification is validated and it is not modified.

#### Recognition of epithelial tissue using the histological ontology

Epithelial tissue is recognised by histologist, biologist and pathologists using commonly light regions as key information since epithelial cells are always found close to these regions. These specialists usually employ images taken with a $40\times$ objective to classify this type of tissue according to the shape of cells—flat, cubic or cylindrical. However, we propose an approach to recognise the epithelial tissue on images taken with the $10\times$ objective using the discriminant classes obtained in the preview step, size of light regions, and the histological ontology.

**Figure 3:**
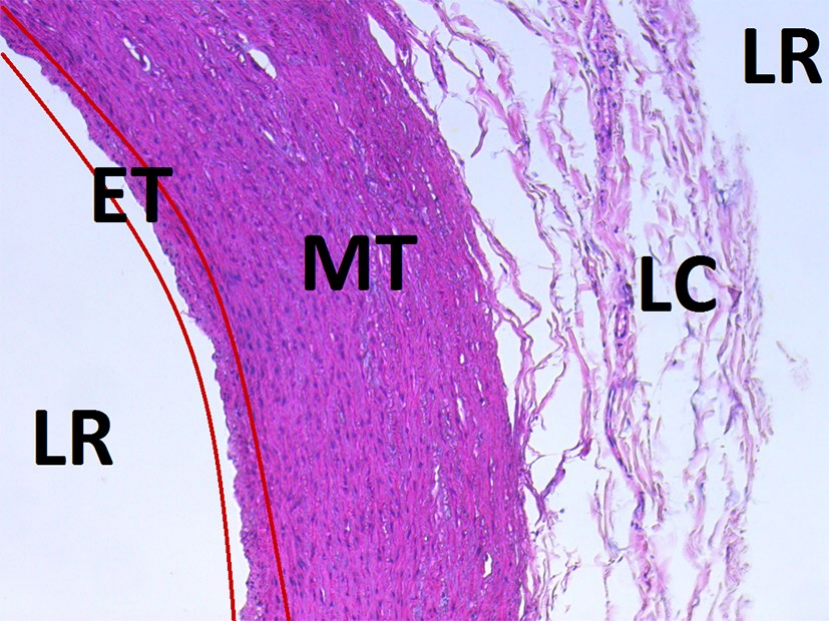
Fundamental tissues in a histological image. *LR* light regions, *LC* loose connective tissue region, *MT* muscle tissue region, *ET* epithelial tissue region (between red lines marked).

Firstly, the size of light regions are evaluated to decide if epithelial tissue is present in the image. Taking into account the organs to be recognised—the heart, the muscular artery, the elastic artery and the large vein—there could be a higher chance to find epithelial tissue if the light regions are are big enough, in our case ten or more consecutive blocks. This value was selected heuristically considering different samples and the specific magnification using Eq. (3). Secondly, the light regions’ neighbourhood should contain smooth muscle of the muscular artery, smooth muscle of the large vein or smooth muscle of the elastic artery. This restriction eliminates false positive cases when there are light regions between loose connective tissue or close to the adventitia tunic (see Fig. 3). Thirdly, rules in RDF form are built considering the subject obtained of the discriminant class with higher occurrences; the object *EpithelialLining* which corresponds to the epithelial tissue; and the predicates *someValuesFrom* and *subClassOf*. These rules are used to make a query with the histological ontology’s reasoner, using SPARQL queries, to obtain a result. The possible answers are the following two cases: (i) the type of epithelial tissue present in the subject, and (ii) an empty result which means that there is a high probability that histological images do not contain epithelial tissue. Finally, blocks over the limit between light region and muscle region are identified as the type of epithelial tissue resulting from the SPARQL query.

3 $$\begin{aligned} \mathop {\mathrm{argmin}}\limits _{t \in {\mathbb {R}}} Ae(\rho ) =\sum _{\rho _{i} \in \rho } {\left| \rho _{g} - \rho _{i}(t)\right| }, \end{aligned}$$

where $\rho _{i}(t)$ is the result obtained automatically when the value *t* is used as input of the algorithm, and $\rho _{g}$ is the ground-truth.

## Results

In this section, we discuss the performance of the proposed methods: (i) improving the classification of organs and (ii) recognition of epithelial tissue. We present F-Score obtained with automatic classification and the improvement process, this is the measurement used to assess the response of the proposed approach in the classification of the following classes: “EA” represents the smooth muscle of the elastic artery, “LV” represents the smooth muscle of the large vein, “MA” represents the smooth muscle of the muscular artery, “HE” represents the cardiac muscle of the heart, “LC” represents the loose connective tissue (i.e. veins, arteries and the heart), and “LR” represents the light regions.

### Experimental setup

Helthy tissue samples from organs were stained with Hematoxylin and Eosin and Masson’s trichrome using a laboratory protocol to control the process. The image capture protocol was defined for settling microscope configuration, software configuration, sample manipulation and image capture process in order to control variability. A dataset of 20 images obtained from tissue samples of various organs and persons, and acquired at 10*x* objective. Images were separated from the start into training and validation ($70\%$, and $30\%$, respectively) and subsequently tiled up to extract 6000 blocks in total. Each one of these samples were manually labelled by histology experts. The group of histology experts consisted of six members of the research group Teblami, from the University of Valle. The histological images were acquired with a Leica *DM750-M* microscope with a resolution of $2048\times 1536$ pixels and stored in *PNG* format. The microscope has an eyepieces with a magnification factor of $10\times$ and a field of view of 20 obtaining 100 end magnification for a $10\times$ objectives. We have made the dataset publicly available at http://biscar.univalle.edu.co/?page_id=1003. Algorithms were implemented in *C++*, using the *CImg* and *libSVM* libraries in a *8-cores* and *8Gb* *RAM computer*.

### Improving organ classification using the histological ontology

Four specific rules are used to illustrate and evaluate our process for improving organ classification: has the heart presence of the elastic artery? if the result is an empty answer it means the heart does not have presence of the elastic artery. Then, blocks which were classified with the elastic artery should be reclassified.

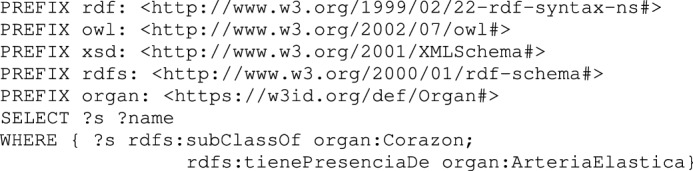



Has the heart presence of the large vein? if the result is an empty answer it means the heart does not have presence of the large vein. Then, blocks which were classified with the large vein should be reclassified.

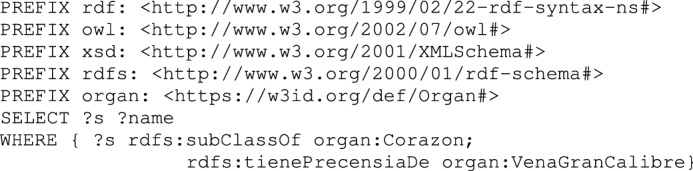



Has the large vein presence of the large vein? if the result is an empty answer it means the large vein does not have presence of the elastic arteries. Then, blocks which were classified with the elastic arteries should be reclassified.

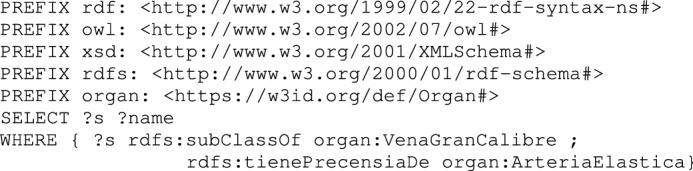



Has the large vein presence of the muscular artery? if the result is an empty answer it means the large vein does not have presence of the muscular artery. Then, blocks which were classified with the muscular artery should be reclassified.

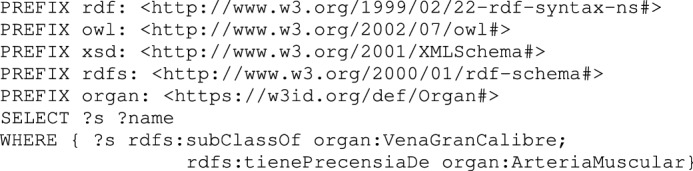



Although arteries and veins are anatomically close, each one accompanied by its concomitant, they are evaluated separately when histological samples are obtained. This part is assessed using the ground-truth classification. According to our experiments, the new label for the refinement process will be the loose connective tissue based on the analysis of false positive in classification process.

**Figure 4:**
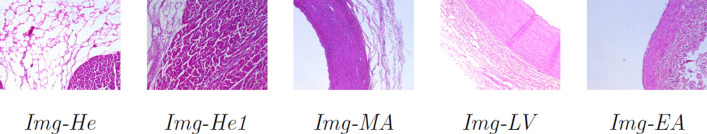
Histological images. *Img-He* and *Img-He1* are the heart images; *Img-MA* is a the muscular artery image; *Img-LV* is a the large vein image; and *Img-EA* is an the elastic artery images.

**Figure 5:**
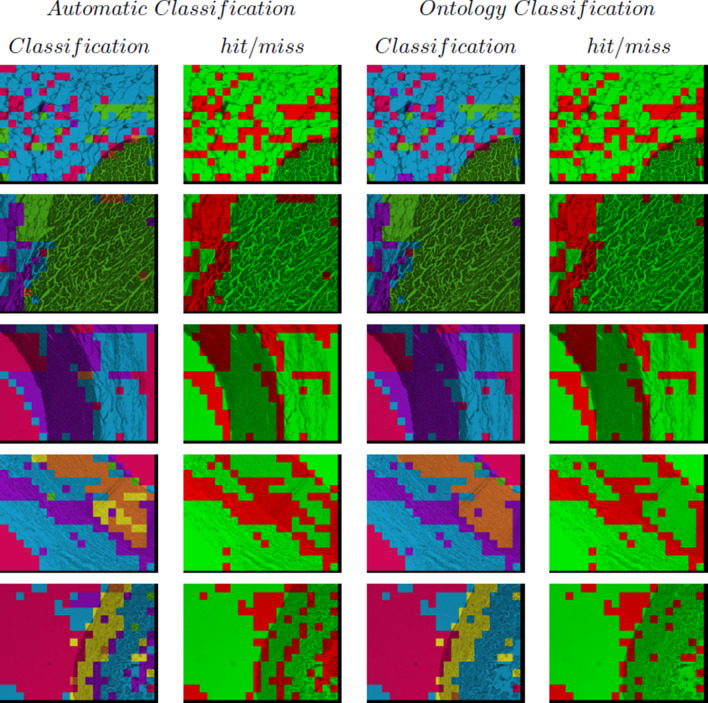
Results of classification using the Ontology. In the first column, automatic classification. In the second column, hits and misses of automatic classification. In the third column, classification using the Ontology. In the fourth column, hits and misses of the Ontology-based classification. In each row a histological image is represented, from top to bottom: *Img-He*, *Img-He1*, *Img-MA*, *Img-EA*, and *Img-LV*.

A selected set of histological images is presented in Fig. 4 and the results of the classification, both initial and using the Ontology, with their hits and misses are included in Fig. 5. Each class has a distinctive colour as follows: (i) cardiac muscle of the heart with green, (ii) loose connective tissue with blue, (iii) smooth muscle of the muscular artery with violet, (iv) smooth muscle of the large vein with yellow, (v) smooth muscle of the elastic artery with orange, and (vi) light regions with fuchsia. On the other hand, hits and misses are represented in green and red colours, respectively. It can be observed that hits increase whilst misses decrease when the Ontology is used to review the initial classification.

The restrictions modelled in the ontology corrects block misclassification. The reclassification of blocks appears in the followings two cases: (i) the confidence of the classification is not high enough to give a verdict about the presence of a tissue, this case takes place when more than one tissue is presented in a block, and (ii) the classifier finds a suitable class, meaning that this tissue is found in that block, but the Ontology says that it is not possible and its presence is discarded.

### Recognition of epithelial tissue

Four specific rule cases are used to illustrate and evaluate our method to recognise epithelial tissue by using the Histological Ontology: what type of epithelium is present in the heart? the type of epithelial tissue present in the heart or an empty result which means that there is a high probability that histological images do not contain epithelial tissue.

$$\hbox {Subject}= \hbox {the heart}$$

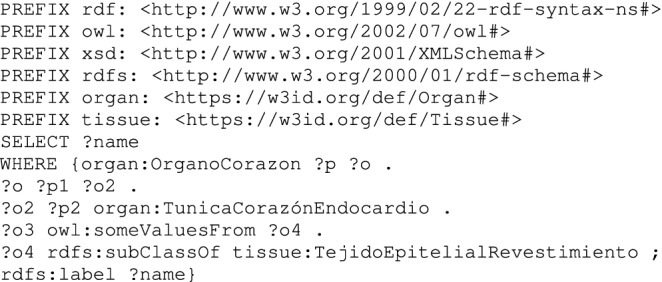



What type of epithelium is present in the elastic artery? the type of epithelial tissue present in the elastic artery or an empty result which means that there is a high probability that histological images do not contain epithelial tissue.

$$\hbox {Subject}= \hbox {the elastic artery}$$.

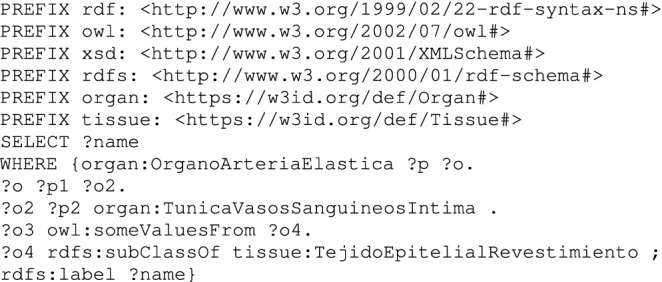



What type of epithelium is present in the muscular artery? the type of epithelial tissue present in the muscular artery or an empty result which means that there is a high probability that histological images do not contain epithelial tissue.

$$\hbox {Subject}= \hbox {the muscular artery}$$

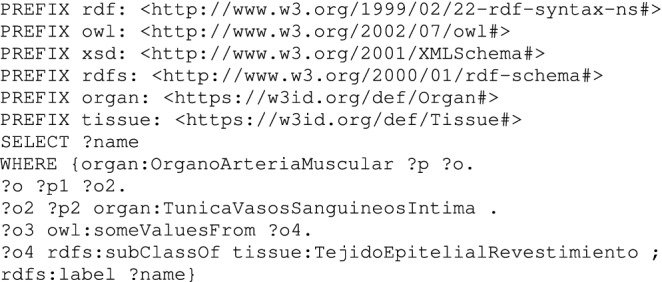



What type of epithelium is present in the large vein? the type of epithelial tissue present in the large vein or an empty result which means that there is a high probability that histological images do not contain epithelial tissue.

$$\hbox {Subject}= \hbox {the large vein}$$

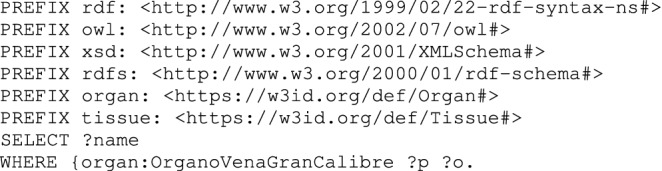



**Figure 6:**
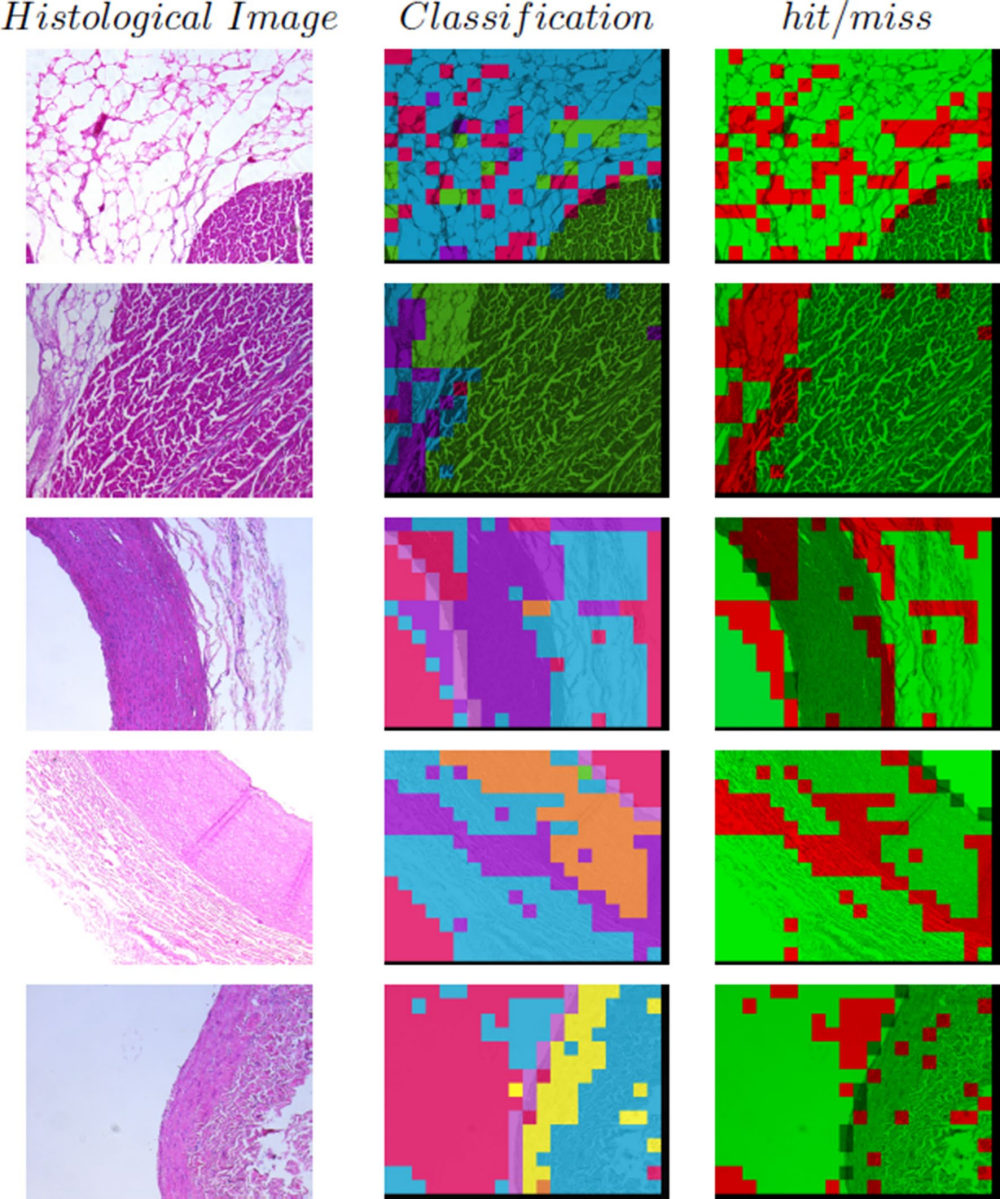
Results of epithelial tissue recognition in a histological image. In the first column, histological images. In each row from top to bottom: *Img-He* and *Img-He1* represent the heart images; *Img-MA* represents the muscular artery image; *Img-EA* represents the elastic artery images; and *Img-LV* represents the large vein image. In the second column, automatic classification. In the third column, hits and misses of automatic classification.

A selected set of histological images, recognition of epithelial tissue results and their hits and misses are included in Fig. 6. Each class is depicted with a distinctive colour as follows: (i) cardiac muscle of the heart with green, (ii) loose connective tissue with blue, (iii) smooth muscle of the muscular artery with violet, (iv) smooth muscle of the large vein with yellow, (v) smooth muscle of the elastic artery with orange, (vi) light regions with fuchsia, and (vii) flat simple epithelial tissue with pink. On the other hand, hits and misses are represented in green and red colours, respectively.

**Figure 7:**
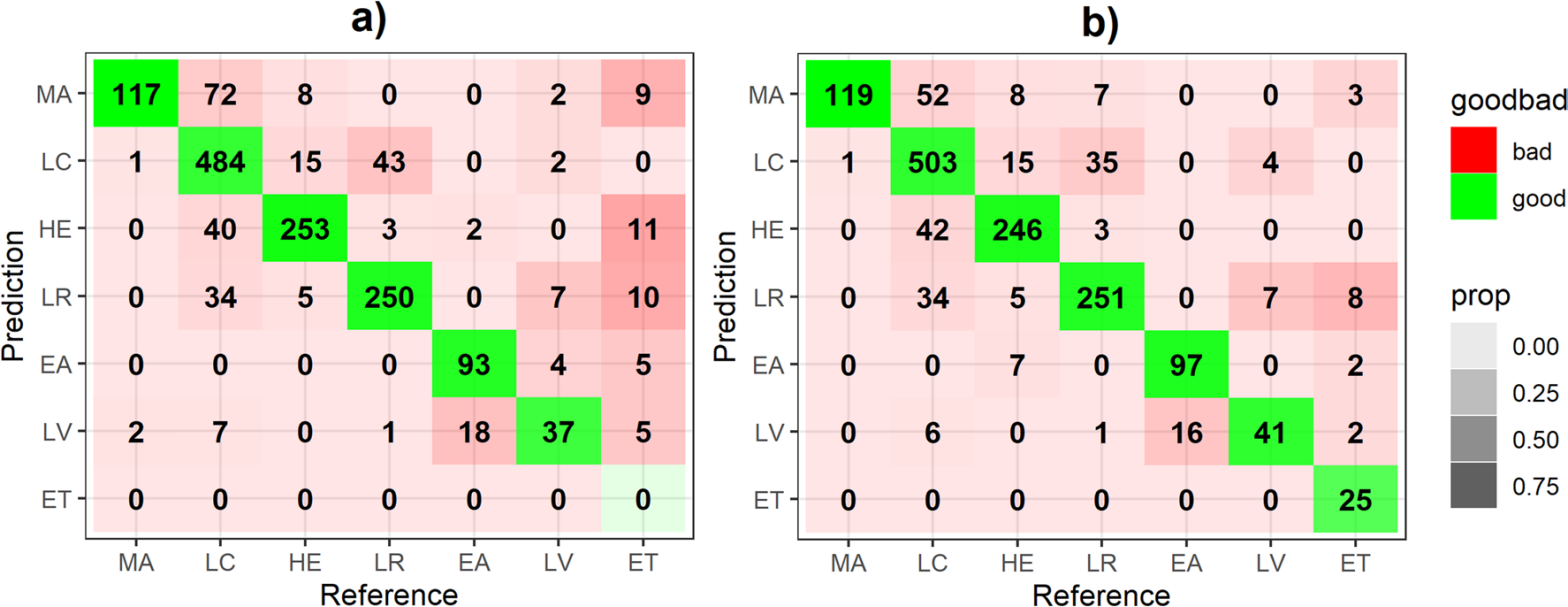
Confusion Matrix. (a) Corresponds to the automatic classification. (b) Corresponds to the ontology-based classification.

**Figure 8:**
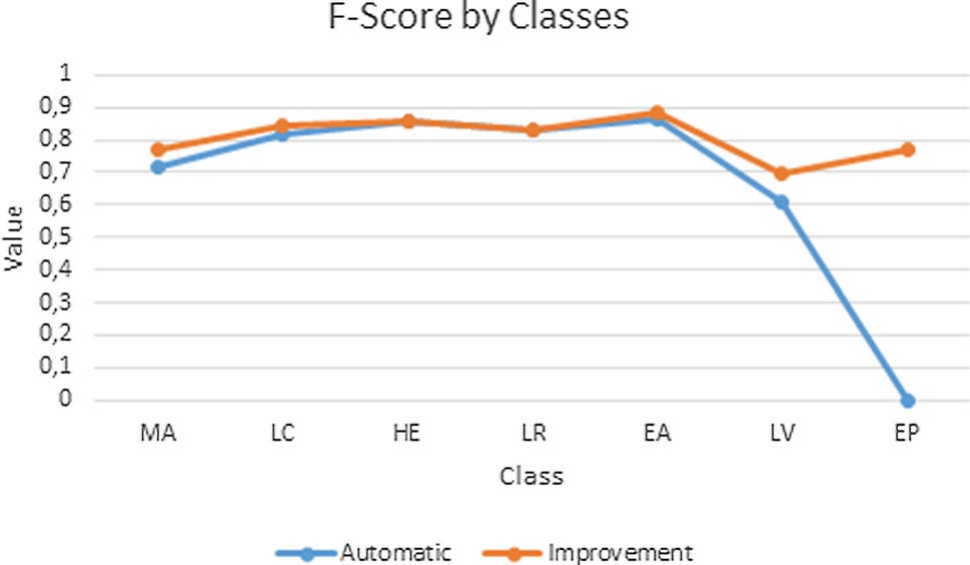
F-Score obtained with automatic classification and Ontology-based process. *MA* smooth muscle of the muscular artery, *HE* cardiac muscle of the heart, *EA* smooth muscle of the elastic artery, *LV* smooth muscle of the large vein, *EP* epithelial tissue, *LC* loose connective tissue—vein, arteries and the heart—and *LR* light areas.

The confusion matrix of both automatic and Ontology-based classification are presented in Fig. 7. Fig. 8 contains a graphical representation of F-Score measures obtained with automatic identification and the Ontology-based processes—organ classification and epithelial tissue recognition—in the set of test images. The obtained results yield between 0.769 and 0.886 F-Score using our proposal. The highest F-Score is obtained for smooth muscle of the elastic artery, since the second step of the classification in the cascade SVM process considers only two possible classes. Although improvement rates of applying Ontology-based classification are uneven among images and classes, the F-Score is higher in all cases. Additionally, using our proposal we can recognise epithelial tissue, what was impossible to do using only image processing. This fact also causes an increase in the F-Score of images containing epithelial tissue. The worst F-Scores are achieved by large vein due to its high similitude with elastic artery according to texture information.

### Automatic classification using a histological ontology vs deep learning approach

**Figure 9:**
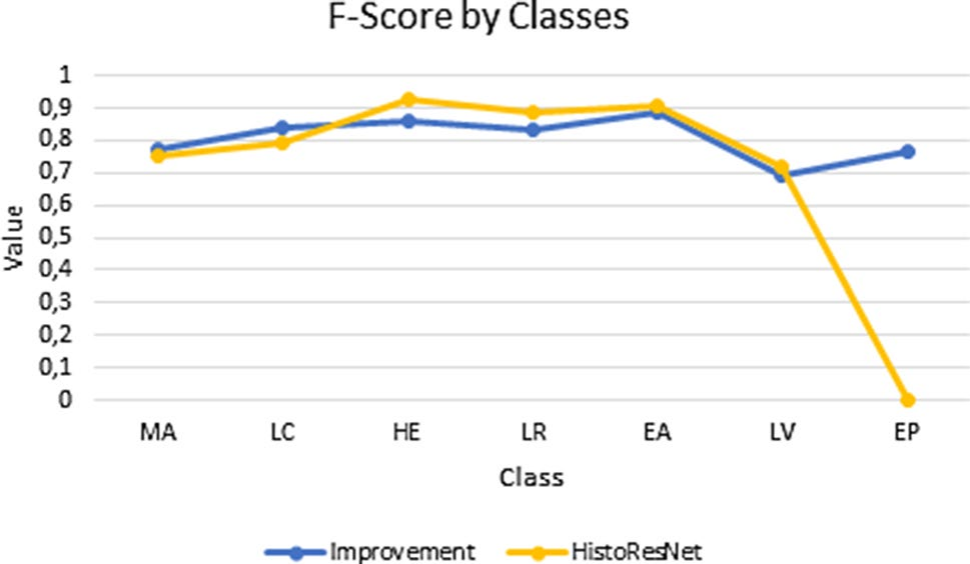
F-Score obtained with ontology-based process and HistoResNet. *MA* smooth muscle of the muscular artery, *HE* cardiac muscle of the heart, *EA* smooth muscle of the elastic artery, *LV* smooth muscle of the large vein, *EP* epithelial tissue, and *LC* loose connective tissue—vein, arteries and the heart—and LR represents the light areas.

In order to compare our ontology-based proposal with Deep Learning techniques, we compared our results with HistoResNet approach presented in Ref.^15^. Figure 9 contains a graphical representation of F-Score measures obtained with the Ontology-based processes and HistoResNet proposal in the set of test images. The highest F-Scores for the smooth muscle of the muscular artery, the loose connective tissue, and the epithelial tissue are obtained with the ontology-based process. On the other hand, HistoResNet has the best F-scores for the cardiac muscle of the heart, the light region, the smooth muscle of the elastic artery, and the large vein. However, the distance between the two methods are not statistically significant difference $(min=0.01; mean=0.14; max=0.76)$, the biggest difference is in the epithelial tissue with 0.77. Both methods coincide with the worst F-Score for the large vein due to its high similitude with elastic artery. Of note, the epithelial tissue is not recognised in images with $10\times$ magnification which were used to train the CNN methods.

### Automatic classification using a histological ontology vs ensemble of classifiers in a late-fusion fashion

**Figure 10:**
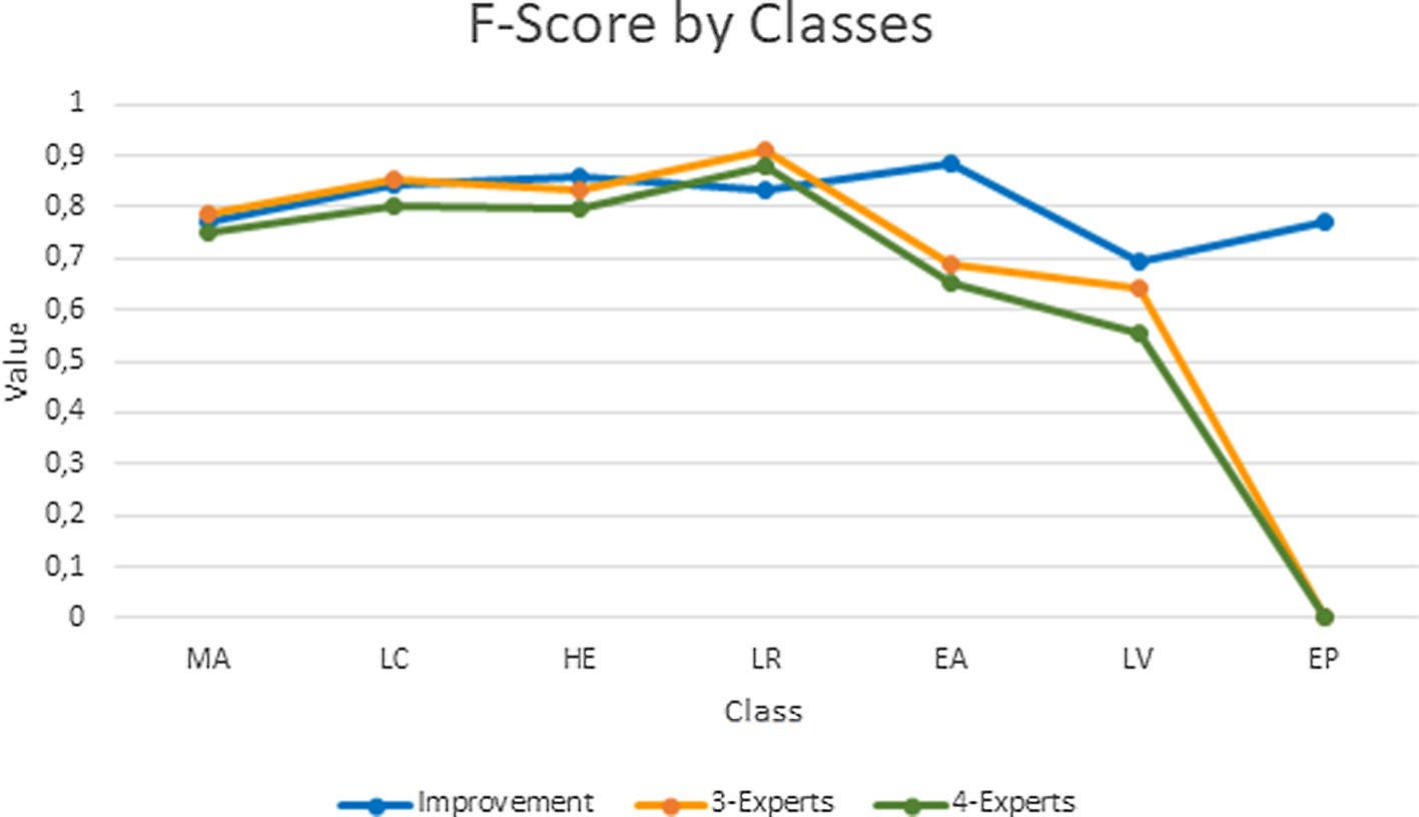
F-Score obtained with the ontology-based process and the ensemble of classifiers. *MA* smooth muscle of the muscular artery, *HE* cardiac muscle of the heart, *EA* smooth muscle of the elastic artery, *LV* smooth muscle of the large vein, *EP* epithelial tissue, and *LC* loose connective tissue—vein, arteries and the heart—and *LR* light areas.

We compared our ontology-based proposal against the HistoResNet, HistoVGG19, HistoVGG16, and HistoInception transfer learning approaches presented in Ref.^15^. We considered an ensemble of classifiers in a late-fusion fashion through majority voting. Firstly, we used majority voting to predict the final verdict, the most frequent class was output. After, we used weighted majority voting for breaking ties. In this case, the HistoResNet had the highest weight since it performed the best in previous experiments^15^. We proposed a three-experts and four-experts considering the top three and four performances^15^—HistoResNet, HistoVGG16, HistoVGG19, and HistoInception. Figure 10 depicts the F-Score values obtained by our ontology-based proposal vs the ensemble of classifiers.

The highest F-Scores for the smooth muscle of the cardiac muscle of the heart, the smooth muscle of the elastic artery, the smooth muscle of the large vein, and the epithelial tissue were obtained by our ontology-based strategy. The mixture of three-experts had the best F-scores for the smooth muscle of the muscular artery, loose connective tissue, and light areas. The biggest difference between our proposal and the ensembles was for the smooth muscle of the elastic artery with 0.20 for three-experts and 0.23 for four-experts. The lowest F-Scores obtained with the ensemble of classifiers were for the smooth muscle of the elastic artery and the smooth muscle of the large vein, outcome which matches previous results^15^. Of note, the epithelial tissue is not recognised in images with $10\times$ magnification which were used to train the CNN methods.

## Discussion

The results obtained cannot be compared to other approaches in the state-of-the-art because, although there are other solutions for include ontologies in the fields of image analysis, these other works addressed different challenges, such as annotation^4^, histopathological images^4^ and^5^, or they used taxonomies^6^ which is limited in inferencing potential due to lack of relational expressiveness about its content. However, we compared our results with our last approach without using the histological ontology and an ensemble of classifiers in a late-fusion fashion. To the best of our knowledge, this is the first time that the problem of the classification of the fundamental tissues and organs, obtained by computer vision techniques, by using the knowledge contained in a histological ontology has been addressed, obtaining the best accuracy up to date.

Two trends were observed regarding the classification of the six classes. On the one hand, Large Vein exhibited the least F-score compared to the rest of the classes, what can be due to the similarity with elastic artery according to texture information. It is important to highlight that this situation is not limited to our proposal as it was already observed during the manual annotations as well. On the other hand, it is evident the improve of epithelial tissue classification with a F-Score of 0, 769.

## Conclusions

In this paper, we presented a process that improves the classification of the healthy fundamental tissues and organs, obtained by computer vision techniques, by using the knowledge contained in a histological ontology. Our method enables to obtain more consistent information and to reduce the error and uncertainty through corroboration and verification, by comparing every analysis of the different data sources separately.

We have improved the initial classification based on computer vision, by using the knowledge contained in a histological ontology. With the proposed method, between 0.769 and 0.886 F-Score were obtained. Additionally, the improved process increases F-Score of the initial classification between 0.003% and $0.769\%$ according to the histological image and its automatic classification. The results revealed that the proposed method increases F-Score in all cases.

We compared our methodology with a CNN proposal called HistoResNet and an ensemble of classifiers in a late-fusion fashion (HistoResNet, HistoVGG16, HistoVGG19, and HistoInception) obtaining similar results in most of the classes except for the epithelial tissue, where our proposal demonstrates its superiority because it is capable of recognising this tissue whiles the CNN and the ensemble classifier are not.

Additionally, we have introduced a new method to recognise the epithelial tissue based on the histological ontology classification. We have concluded that the improved classification using a histological ontology enables us to identify the epithelial tissue in images with $10\times$ magnification whereas images with $40\times$ magnification are not always available to recognise areas of epithelial tissue.

Besides, the use of an Ontology, comparing with the use of taxonomies^6^, allows to obtain and infer more information of histological knowledge such as a system, a composition, but also as structures, relations, regions, layers, sectors, tissues and cells.

The ontology-based classification includes histology information additional to texture features from image classification. This improve results and make the method more robust and complete. However, this proposal is limit to texture features in this stage. Additionally, as another contribution, we have created and made publicly available a dataset consisting of 6000 blocks that can be used to validate the results obtained in our work.

In the future, we will extend this proposal by working in the following two lines: (i) exploring our ontology-based methodology using CNN strategies instead of SVM; (ii) exploring an adaptive block size to reduce the misclassification in edges areas; (iii) in this work, we compared our proposal against ensembles of CNNs. We used majority and weight voting. We plan to consider other ensembles strategies. (iii) including global features, spatial and relational features, on the classification performance to include more discriminant information among classes. (iv) to use macro-circulation identification to recognise micro-circulation organs; and (v) to apply the ontology-based classification process to different problems in medicine and industry. A re-definition of ontological rules to the specific context are needed considering knowledge information depends on the application domain or particular subject area.
